# Distinct Neural Networks Relate to Common and Speaker-Specific Language Priors

**DOI:** 10.1093/texcom/tgaa021

**Published:** 2020-05-29

**Authors:** Leon O H Kroczek, Thomas C Gunter

**Affiliations:** Department of Clinical Psychology and Psychotherapy, University of Regensburg, Regensburg 93053, Germany; Department of Neuropsychology, Max Planck Institute for Human Cognitive and Brain Sciences, Leipzig 04103, Germany

**Keywords:** neuroimaging, prediction, speaker identity, syntax, top-down

## Abstract

Effective natural communication requires listeners to incorporate not only very general linguistic principles which evolved during a lifetime but also other information like the specific individual language use of a particular interlocutor. Traditionally, research has focused on the general linguistic rules, and brain science has shown a left hemispheric fronto-temporal brain network related to this processing. The present fMRI research explores speaker-specific individual language use because it is unknown whether this processing is supported by similar or distinct neural structures. Twenty-eight participants listened to sentences of persons who used more easy or difficult language. This was done by manipulating the proportion of easy SOV vs. complex OSV sentences for each speaker. Furthermore, ambiguous probe sentences were included to test top-down influences of speaker information in the absence of syntactic structure information. We observed distinct neural processing for syntactic complexity and speaker-specific language use. Syntactic complexity correlated with left frontal and posterior temporal regions. Speaker-specific processing correlated with bilateral (right-dominant) fronto-parietal brain regions. Finally, the top-down influence of speaker information was found in frontal and striatal brain regions, suggesting a mechanism for controlled syntactic processing. These findings show distinct neural networks related to general language principles as well as speaker-specific individual language use.

## Introduction

Language is a dynamic system. From birth on, we learn about language by using it and listening to it. Every uttered or perceived sentence may be viewed as a training stimulus shaping our language system (MacDonald 2013). After a lifetime of language use and numerous encounters with different speakers, we have a solid representation of language use on a population level. The reflection of this lifetime experience with language can be thought of being represented in general language priors (Levy 2008; Gibson et al. 2013). Such priors capture fundamental principles of a given language and allow for a fast and efficient processing of language (Hale 2001; Smith and Levy 2013; Kuperberg and Jaeger 2015 for an overview). On the downside, however, priors may become inefficient when they do not match the actual language input (Fine et al. 2013).

Syntactic structure may be a relevant example for this, because even languages with a flexible word order (e.g., German) show a dominance of a particular type of syntactic word order (i.e., subject-initial structure) that is also reflected in reduced processing costs and neural effort in comprehension of these structures compared with less frequent and more complex structures (i.e., object-initial structures). While such general language priors may account for a population-wide use of language, they may be less suited in the case of an individual speaker with a distinct pattern of language use.

There are several examples where speakers deviate substantially from the “general” pattern of language use. For instance, regional variations of syntactic structure have been reported for different dialects (cf. Fanselow et al. 2005). Crucially, adaptation to language variations is possible (Fine and Jaeger 2013, 2016; Fraundorf and Jaeger 2016; Ryskin et al. 2017). Fanselow et al. (2005) found that speakers, who were not familiar with a particular dialectal syntactic variation, started to adopt this previously unknown variation after short exposure.

Variations may also occur on the next level of detail, relating to individual persons. Some interlocutors might use more difficult structures than the average person, whereas other interlocutors might be more error-prone, for example, when they speak in a nonnative language (i.e., Hanulíková et al. 2012). This individual variance is in contrast to the fundamental principles observed across speakers, and one might expect that language comprehension for such deviating speakers is slow and effortful. However, recent experiments show that listeners are highly successful in adapting to the individual language use of a particular speaker and that language processing might be altered accordingly (Hanulíková et al. 2012; Kroczek and Gunter 2017). Together with evidence on contextual constraints on language processing (Tanenhaus et al. 1995), these findings suggest that speaker identity might serve as a context that allows to generate particular syntactic expectations (Brown-Schmidt et al. 2015; see also Kuperberg and Jaeger 2015). This has been shown both for semantic and syntactic features of language (Van Berkum et al. 2008; Hanulíková et al. 2012; Kamide 2012; but see Liu et al. 2017). We additionally know that adaptation happens quite rapidly (even after a few sentences; Fine et al. 2013; Farmer et al. 2014) and seems to be robust and long lasting (up to at least 9 months) once it has been established (Kroczek and Gunter 2017). These findings suggest the existence of speaker-specific language representations that can be used to enhance language processing.

One intriguing question relates to the brain basis of these general and individual aspects of language understanding. Syntactic language processing has been related to a functional and anatomical circuit in frontal and temporal brain areas of the left hemisphere (Friederici 2012; Hagoort and Indefrey 2014). Syntactic processing has been frequently investigated using scrambling constructions (i.e., object-initial sentences in German) that deviate from basic word orders (subject-initial sentences in German) and which have been related to processing difficulty. This difficulty has been linked to higher working memory demands (i.e., Fiebach et al. 2005; but see Makuuchi et al. 2009). Furthermore, language-specific linearization of hierarchical linguistic dependencies is also thought to play an important role (Grewe et al. 2005). Most fMRI studies on scrambling constructions in German have shown activity typically in the left IFG (BA44/45: Röder et al. 2002; Fiebach et al. 2005; BA44: Bornkessel et al. 2005; Grewe et al. 2005, 2006; Friederici et al. 2006; Bornkessel-Schlesewsky et al. 2009). Also, posterior temporal cortex has been reported for syntactic processing (Röder et al. 2002; Friederici et al. 2003b; Bornkessel et al. 2005; Friederici 2011; Segaert et al. 2012).

While the functional specificity and neural organization of syntactic processing are a matter of ongoing debate (Fedorenko and Thompson-Schill 2014), little work has been conducted to investigate neural mechanisms of speaker-specific use of syntactic structures. It remains unknown whether speaker-specific processes might invoke similar or distinct neural mechanisms as general language priors. One could argue that both represent the same type of information and therefore should be processed in the same brain structures. However, it might be inefficient to adapt an existing language prior just for one speaker (who has been known for a couple of sentences). Therefore, language priors and speaker-specific language use might be represented in distinct systems.

So far, this issue has not been tested yet for syntactic processing. On the speaker side, a lot of work has been done to identify systems that underlie speaker recognition (Belin and Zatorre 2003; Von Kriegstein et al. 2003; Belin et al. 2004; Perrodin et al. 2015), but we do not know how this basic processing leads to more high-level representations such as used in language. On the language side, brain processes underlying linguistic function have been well established (Goucha and Friederici 2015; Zaccarella et al. 2017), but we do not know whether this reflects general or specific information. In fact, previous studies have confounded these 2 aspects because they typically present complex language stimuli that are also very infrequent in language use.

The current study addresses this question, by investigating neural processing related to speaker-specific use of scrambling constructions in German. For that reason, we implemented a paradigm where listeners are exposed to a complex speaker who mainly uses scrambled constructions (object-initial sentences) as well as an easy speaker who mainly uses basic syntactic structures (subject-initial sentences). This allows us to disentangle processing of syntactic complexity (priors) and processing of speaker-specific language use. Apart from possible distinct neural representations, the existing literature suggests an interaction between speaker-specific information and sentence comprehension via top-down mechanisms (Hanulíková et al. 2012; Brothers et al. 2019). The investigation of such top-down influences is another goal of the present study. For that reason, we included another type of sentence stimuli, the so-called probe stimuli, which are ambiguous with respect to their actual syntactic sentence structure but allow recognizing speaker identity. By measuring neural and behavioral responses to these stimuli, we can test the top-down influence of speaker information in the absence of syntactic structure information.

## Materials and Methods

### Participants

Thirty-three participants completed the experiment. Five participants were excluded from the analysis as their behavioral data were lost due to technical problems (twenty-eight remaining participants: mean age = 25.64 years, age range = 20–31 years, 14 women and 14 men). Sample size was chosen on the basis of a previous behavioral study using a similar paradigm (Kroczek and Gunter 2017) as well as previous fMRI studies (Snijders et al. 2009; Goucha and Friederici 2015). All participants were right-handed, native German speakers with normal or corrected-to-normal vision. Participants had a mean laterality quotient of 92.96 (SD = 9.00, Oldfield, 1971). None did report a history of neurological or psychiatric disease or any hearing deficits. All gave written informed consent and received 36€ for compensation. Experimental procedures were approved by the ethics committee of the University of Leipzig (159-16/ek-25 042 016).

### Materials

**Figure 1:**
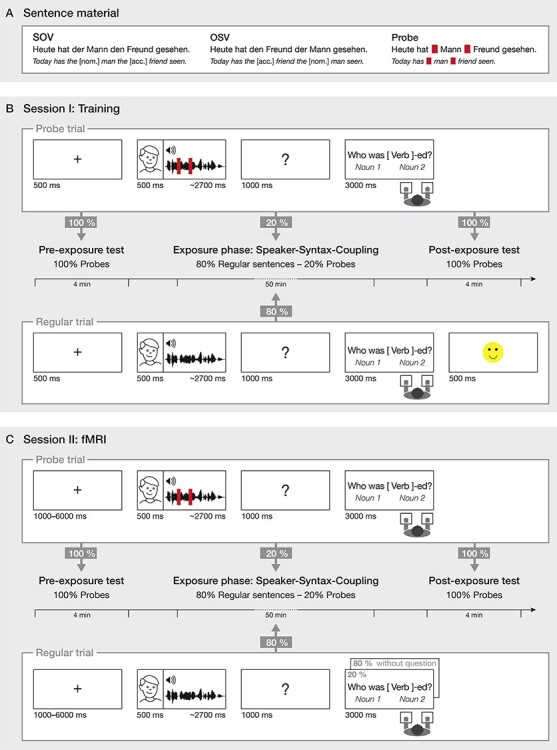
(*A*) Example of the sentence material used in the experiment. Regular sentences had either a Subject-Object-Verb structure or an Object-Subject-Verb structure. In addition, probe sentences were presented, where determiners had been replaced by white noise, thus rendering the sentence ambiguous (white noise indicated in red). (*B*) Experimental procedure and trial structure in the training session. The session started with a preexposure test, where only probe trials of both speakers were presented. This was followed by an exposure phase, where both regular and probe trials were presented. The regular trials established the speaker-syntax coupling as the probabilities of SOV and OSV structures differed on the basis of speaker identity, that is, the SOV-Speaker had a high probability to produce a SOV structure, while the OSV-Speaker had a high probability to produce an OSV structure. Probe trials of both speakers were always ambiguous with respect to syntactic structure due to the white noise manipulation (indicated in red). For both regular and probe trials, a comprehension question was presented, which asked participants to indicate either the subject or the object of the sentence. Feedback (correct/incorrect) was presented only for the regular trials. Finally, a postexposure test was presented with probe trials only. (*C*) Experimental procedure and trial structure in the fMRI session. The procedure was similar to the training session with the following differences: The exposure phase was conducted while participants were inside the MRI scanner (preexposure and postexposure were conducted outside the scanner). For the regular trials, the comprehension question was only presented in 20% of all trials, and no feedback was given. Importantly, the probe trials always included a comprehension question (but no feedback).

The experimental German sentences consisted of a short lead*-*in phrase, two noun phrases and a verb. Sentences had either a subject-initial Subject-Object-Verb (SOV) structure or an object-initial Object-Subject-Verb (OSV) structure (Fig. 1*A*). Sentence structure was defined by the case-marking of the determiners. A nominative determiner specified a subject noun-phrase (e.g., *der Mann*/the[nom.] man), while an accusative determiner specified an object noun-phrase (e.g., *den Freund*/the [acc.] friend). For every noun-noun pair, 2 sentence versions were created with each noun being implemented either as the subject or as the object of a sentence. An original set of 320 noun-noun-verb combinations thus lead to 1280 different sentences.

The stimulus material was spoken by two professional voice actors (female and male) and recorded at 44.1 kHz (Audacity 2.0). In a postprocessing step, a 50 ms period of silence was added at the beginning, at the end and at the onset of the first determiner of every sentence. Sentences were normalized using the Root Mean Square (RMS) of the amplitude. An additional “noise” version was created for every sentence by replacing the determiners with white noise (custom Matlab scripts). In these sentences, the case information of the determiners was missing, and therefore, sentences were ambiguous with regard to their actual syntactic structure. This ambiguity allowed us to use the sentences in probe trials, since they did not contain any information about the syntactic structure (SOV vs. OSV) of a sentence. In such probe trials, the ambiguous sentences were combined with a comprehension question (see below). These probe sentences contrasted to the regular sentences which did not contain noise and in which the syntactic structure became clear through the determiners.

In addition, picture stimuli of a female and a male face were used to present speaker identity earlier to the auditory stimulus. To this purpose, a female and a male face stimuli were taken from the NimStim set of facial expressions (“neutral” and “mouth closed” conditions; Tottenham et al. 2009).

### Experimental Procedure

The experiment consisted of a training session and a fMRI session which took place on 2 consecutive days. For every participant, a randomized list was created for both sessions, and sentence items were randomly assigned into conditions of syntactic structure and speaker identity. Every session consisted of 3 parts: a preexposure test, an exposure phase, and a postexposure test. While the preexposure and postexposure tests were always conducted outside the MRI scanner, the exposure phase in the fMRI session was conducted inside the MRI scanner (see Fig. 1*B* and *C* for a schematic overview). The purpose of the training session was to introduce the specific speaker-syntax coupling to the participants and thereby to allow participants to generate expectations for a particular syntactic structure on the basis of speaker identity (Kroczek and Gunter 2017). The purpose of the fMRI session on the subsequent day was to measure the underlying neural mechanisms of such expectations.

Every session started with a preexposure test and ended with a postexposure test. Each test comprised 20 ambiguous probe trials (10 per speaker). These tests were included in order to measure baseline levels of participants’ expectations before the exposure phase as well as to measure the participants’ expectations after the exposure to the speaker-syntax coupling.

The exposure phase, in contrast, had both probe trials and regular trials (40 probe trials and 140 regular trials per speaker). The actual speaker-syntax coupling was established only via the unambiguous regular trials. This was done by varying the frequency by which a particular speaker produced a SOV sentence or an OSV sentence. In detail, one speaker, the so-called *SOV-Speaker,* produced SOV sentences more frequently than OSV sentences. Whereas, the other speaker, the so-called *OSV-Speaker*, produced OSV sentences more frequently than SOV sentences. The exact ratio of frequent to infrequent sentence structures within speaker differed between training and fMRI session (see Table 1 for an overview on ratios and exact trial numbers). This was done in order to ensure the build-up of speaker-specific expectations in the training session on the one hand and to allow for reliable effect estimation of the infrequent sentence structure condition in the fMRI session on the other hand. In both sessions, ambiguous probe trials were randomly interleaved between the regular trials in order to track participants’ expectations over the course of the sessions. Furthermore, in the fMRI session, the probe trials allowed to measure neural processing when no overt syntactic structure was presented in the sentence stimuli due to the white noise manipulation (see above). Note, that the gender of the SOV- and OSV-Speaker was balanced across participants.

**Table 1:** Trial numbers in the exposure phase and ratio (within speaker) are shown for both speakers (OSV-Speaker, OSV-Speaker), sentence structures (SOV, OSV Probe), and sessions (training, fMRI)

Session	Exposure training			Exposure fMRI		
Structure	SOV trials (percentage within Speaker)	OSV trials (percentage within Speaker)	Probe trials	SOV trials (percentage within Speaker)	OSV trials (percentage within Speaker)	Probe trials
**SOV-Speaker**	128 (91.43%)	12 (8.57%)	40	100 (71.43%)	40 (28.57%)	40
**OSV-Speaker**	12 (8.57%)	128 (91.43%)	40	40 (28.57%)	100 (71.43%)	40

The exposure phase of the fMRI session was conducted in the MRI scanner. Auditory stimuli were delivered over headphones using the MR Confon system. The sound level was individually adjusted for every participant before stimulus presentation. Participants were able to observe the visual stimulation via a mirror integrated in the coil.

Trial structure was similar for each of the sessions (see Fig. 1) and was controlled by the stimulus presentations software Presentation (Neurobehavioral Systems). Every trial started with the presentation of a fixation cross. The duration of the fixation cross was jittered (logarithmic distribution, range = 1–6 s, mean = 2.5 s; training session = fixed 500 ms). Next, the face of the upcoming speaker was presented visually on the screen. After 500 ms, the sentence was presented via headphones while the face of the speaker remained on the screen (mean sentence duration = 2.68 s, SD = 0.21 s). Depending on session (training or fMRI) and trial type (regular or probe), comprehension questions were presented after the sentence stimulus. In the training session, all regular exposure trials were followed by a comprehension question assessing whether the participant had understood the intended sentence structure. On all regular trials, feedback indicating whether the participant’s response had been correct was displayed 500 ms after the response. In the fMRI session, only 20% of the regular trials were presented with a comprehension question. And, critically, the comprehension questions were never followed by feedback. In both sessions, probe trials were always followed by a comprehension question without feedback. In trials where a comprehension task was presented, a question mark appeared on the screen for 1000 ms. This was followed by a comprehension question displayed at the top of the screen and 2 response options that were displayed below the question on the left and right side of the screen. Participants had to respond via a button press with the left or right thumb. In both sessions, questions were displayed for 3000 ms and responses after this period were counted as misses.

In addition, the fMRI session also included 40 null trials where only the fixation cross was presented for a duration of 5 s. Null trials were interleaved throughout the whole experiment as basic control stimuli with the same duration as experimental trials. After every 50th trial, there was a break of 25 s.

### Task

The comprehension question had a very different purpose depending on whether it was presented as part of a regular trial or a probe trial. For regular trials, the task allowed to measure participants’ performance and thereby to ensure that they were focusing on the sentences. For probe trials, however, the task allowed to measure participant’s expectations about the syntactic structure of a sentence in the absence of syntactic structure cues. The questions asked either for the subject of the sentence (e.g., “*Who did* [verb]*?*”) or the object of the sentence (e.g., “Who was [verb]-*ed?*”) and participants had to select one of the two nouns that had been presented in the previous sentence (question type and for regular trials the side of the correct answer were balanced within conditions). This was straightforward in regular trials as the determiners allowed to identify subject and object of a sentence. In probe trials, however, there was no correct answer as the noun phrases were ambiguous toward their subject/object status because the determiners had been replaced by noise. Still, participants were asked to answer the question. The position of the selected noun in the previous sentence in combination with the question type (asking for the subject or the object) allowed us to infer participants’ expectations about the syntactic structure of the sentence. For example, if a question asked for the object and a participant had selected the second noun of the sentence as a response, then the sentence was parsed as a SOV structure, whereas, if the first noun was selected, the sentence was parsed as a OSV structure (and vice versa for a question asking for the subject). Note, that even though probe trials were ambiguous toward their syntactic structure, we still balanced them according to the syntactic structure of the original sentences from which they had been generated. A training session lasted about 55 minutes whereas a fMRI session took approximately 75 min.

### MRI Acquisition

Functional data were acquired on a 3T Skyra MRI scanner (Siemens, Erlangen) using a 20-channel coil (TR = 2 s, TE = 30 ms). One volume comprised 31 slices of 3 mm with a gap of 33% (interleaved ascending acquisition). In-plane resolution was set to 3 × 3 mm with a FoV of 192 mm. For every participant, a fieldmap scan was acquired (TR = 488 ms, TE1 = 4.58 ms, TE2 = 7.04 ms). A T1-weighted anatomical scan was available for every participant. In total, 1528 functional volumes were acquired per fMRI session.

### Preprocessing and Analysis of fMRI Data

Data preprocessing and analysis was done in SPM12 (Wellcome Centre for Human Neuroimaging, http://www.fil.ion.ucl.ac.uk/spm/). Preprocessing followed a standard protocol implemented in SPM. First, slice time correction was performed in order to correct for temporal differences between slices of the same volume (Parker and Razlighi 2019). Then motion correction was applied by spatial realignment to the first volume (Johnstone et al. 2006) and distortion correction was applied using the acquired fieldmap (Hutton et al. 2002). Functional images were normalized to MNI space via segmentation of the coregistered anatomical image. Finally, smoothing was applied using an 8-mm FWHM Gaussian kernel (Mikl et al. 2008). Temporal filtering was performed using a high-pass filter with a cut-off of 128 s (SPM default).

Data analysis was conducted on the whole-brain using random-effects group analysis implemented in the general linear model (GLM). A separate first level GLM was fit to the BOLD signal of each individual participant. These first level models included each 6 experimental regressors (4 regular trials: SOV-Speaker SOV, SOV-Speaker OSV, OSV-Speaker SOV, OSV-Speaker OSV; 2 probe trials: SOV-Speaker, OSV-Speaker). For the regular trials, events were defined as the onset of the first determiner as this was the time point where the syntactic structure became clear. Duration was defined as the time between onset of the first determiner and sentence end. For the probe trials, events were defined as the onset of the speaker’s face on the screen as this was the time point where the information about the upcoming speaker was revealed. Duration was defined from face onset to sentence end. Furthermore, a regressor related to the null events was included with event length as duration. Additionally, regressors-of-no-interest were related to fixation crosses, question marks, task presentation and breaks. These 11 regressors were convolved with the canonical hemodynamic response function implemented in SPM. The first level models also included the translational and rotational movement parameters from the motion correction step in the preprocessing. For every participant, the 6 betas corresponding to the experimental conditions of the first level were each contrasted against the beta of the null event regressor resulting in 6 condition contrast images per participant. These contrast images were then modeled as fixed effects in the second-level analysis, while individual subject regressors were entered as random effects.

Two separate second level analyses were performed to accommodate the different nature of regular and probe events. The first random effects group model included the 4 contrast images related to the regular trial conditions of each participant as well as subject-specific random effects. The experimental conditions were modeled using the factors *Speaker* (SOV-Speaker vs. OSV-Speaker) and *Structure* (SOV vs. OSV). Similarly, the second random effects group model included the 2 contrast images related to the probe trial conditions of each participant as well as subject-specific random effects.

### Behavioral Analysis

Behavioral analysis was conducted in the R environment (R Core Team 2016) using mixed-effect models on participant’s responses in probe and regular trials.

Task performance in the regular trials was evaluated with respect to response types (correct vs. incorrect; logit mixed-effect model to account for the categorial nature of the data, Jaeger 2008) and with respect to reaction times (linear mixed-effect model). Reaction times were log-transformed for analysis to account for nonnormal distributions (Wagenmakers and Brown 2007). Both models contained fixed effects for the factors *Structure* (sum coded: SOV = 1, OSV = −1) and *Session* (sum coded: Training session = 1, fMRI session = −1). A full random effects structure was implemented that included random intercepts for every participant and item as well as random slopes by participant for *Structure*, *Session* and the interaction of both (Barr et al. 2013; but see Matuschek et al. 2017).

Responses in the preexposure and postexposure phase were modeled with fixed effects for the factors *Speaker* (sum coded: SOV-Speaker =1, OSV-Speaker = −1) and *Test Position* (Levels: Test Pre-Training, Test Post-Training, Test Pre-fMRI, Test Post-fMRI; treatment coded with Pre-Training as baseline, resulting in 3 contrasts). The maximal random effects structure for which convergence was reached included random intercepts by subjects, random intercepts by items as well as random slopes for *Speaker* by subjects.

## Results

### Behavioral Data

#### Task Performance in Regular Trials

**Figure 2:**
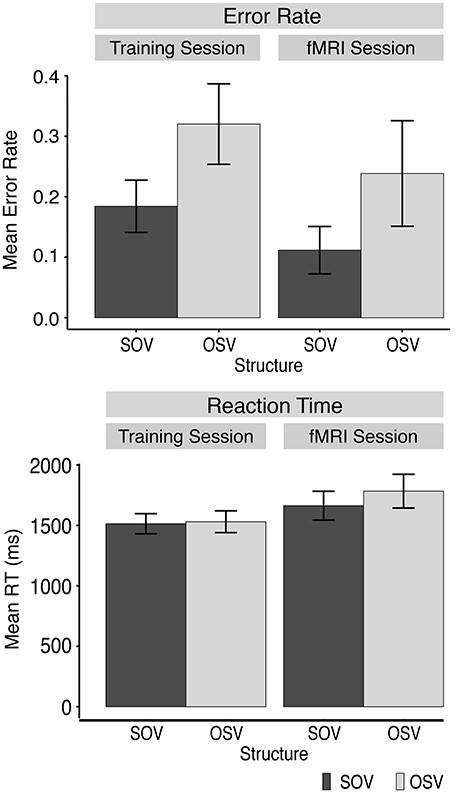
Task performance in regular trials with respect to error rate (top) and reaction times (bottom). Bars show performance for SOV and OSV structure sentences in the training session and the subsequent fMRI session. Error bars reflect 95% confidence intervals over the means of participants.

Participants’ performance in the regular trials of the exposure phase was analyzed with regard to error rate and reaction times using mixed-effects models (Fig. 2). Due to the small number of trials in the infrequent structure conditions (only 8 trials with comprehension questions in the [SOV-Speaker OSV] and [OSV-Speaker SOV] condition in the fMRI session), the models did not include the factor Speaker.

The model of participants’ responses revealed a main effect of *Structure, χ*^2^ = 83.654, *P* < 0.001, with increased errors for the OSV sentences compared with SOV sentences ($\hat{\beta}$ = −0.428, SE = 0.068), and a main effect of *Session, χ*^2^ = 24.357, *P* < 0.001, with decreased error rates in the fMRI session compared with the training session ($\hat{\beta}$ = 0.325, SE = 0.063). These effects persisted in a speed-accuracy analysis (see Supplementary Figs S1–S5).

The model of the reaction times revealed a main effect of *Structure, χ*^2^ = 6.342, *P* = 0.010, with increased reaction times for OSV structures compared with SOV structures ($\hat{\beta}$ = −0.02, SE = 0.004), a main effect of *Session, χ*^2^ = 18.979, *P* < 0.001, with increased reaction times in the fMRI session compared with the training session ($\hat{\beta}$ = −0.059, SE = 0.013), and an interaction of *Structure x Session,* χ^2^ = 11.721, *P* < 0.001, with an increased difference between SOV and OSV structures in the fMRI session compared with the training session ($\hat{\beta}$ = 0.013, SE = 0.004).

In summary, these results demonstrate that comprehension performance was reduced for OSV sentences compared with SOV sentences. The difference in reaction times as a function of session is most likely due to the fact that comprehension questions were presented randomly in only 20% of the trials in the fMRI session, but in all trials in the training session.

#### Probe Trials: Build-Up of Syntactic Expectations

Participant’s responses in the probe trials of the preexposure and postexposure phase of both sessions were analyzed to assess whether participants would successfully generate expectations on the basis of speaker information.

The model revealed a main effect of *Speaker, χ*^2^ = 33.963, *P* < 0.001, and an interaction of *Speaker x Test Position, χ*^2^ = 139.599, *P* < 0.001. There was an increased difference between SOV-Speaker and OSV-Speaker after exposure to the syntax-speaker coupling compared with baseline before the exposure to the syntax-speaker coupling. This could be demonstrated both at the end of the training session, $\hat{\beta}$ = 1.363, *Z* = 8.944, *P* < 0.001, and at both tests of the fMRI session (Pre-fMRI: $\hat{\beta}$ = 1.312, *Z* = 8.567, *P* < 0.001; Post-fMRI: $\hat{\beta}$ = 1.694, *Z* = 10.570, *P* < 0.001).

**Figure 3:**
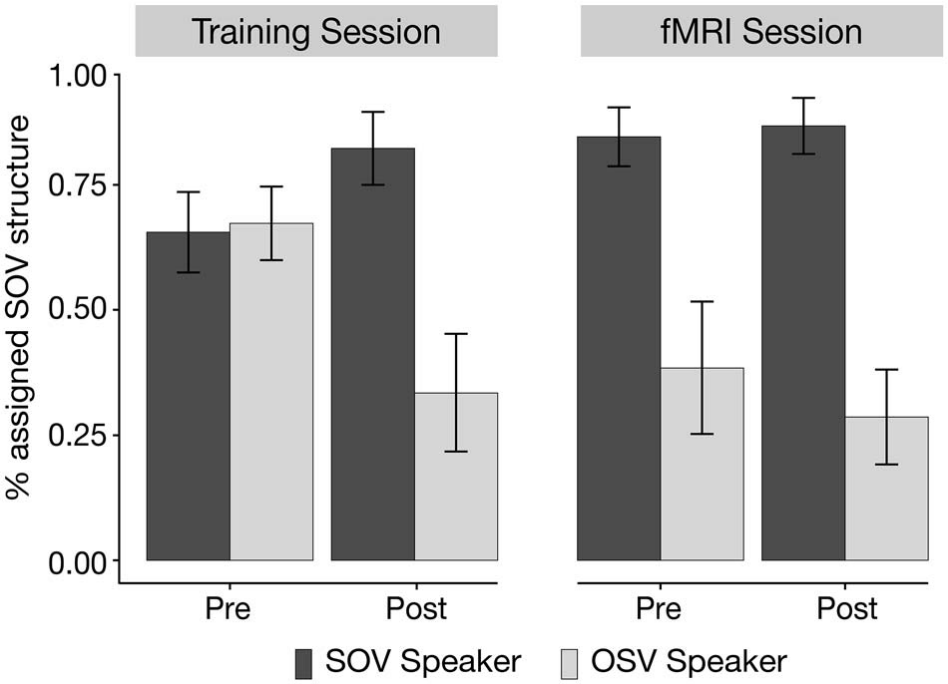
Responses in the ambiguous probe trials of the preexposure and postexposure phase. Bars show the percentage of assigned SOV structures for a particular speaker (SOV-Speaker in dark, OSV-Speaker in light) in the Training session and in the subsequent fMRI session. Error bars reflect 95% confidence intervals over the means of participants.

In line with previous findings (e.g., Kamide 2012; Fine et al. 2013; Kroczek and Gunter 2017) these data demonstrate that participants successfully adapted their syntactic expectations to the language use of a particular speaker (see Fig. 3). Importantly, these expectations were maintained throughout the fMRI session. The results were further supported by an incremental build-up of expectations that was observed in the exposure phase of the training session (see Supplementary Figs S1–S5).

### fMRI Data: Regular Trials

#### Main Effect of Syntactic Complexity

**Figure 4:**
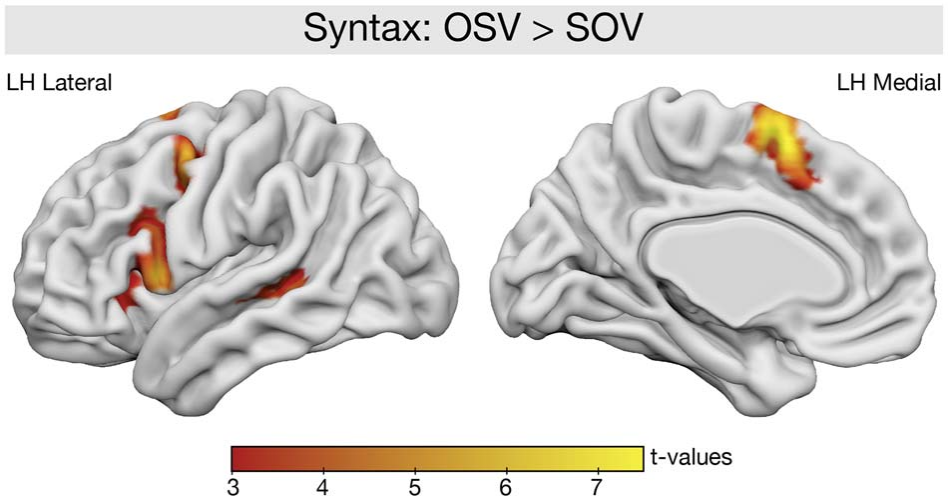
Main effect of syntactic complexity. Significant clusters with increased activation for OSV structures compared with SOV structures plotted on the brain surface. All results are FWE-corrected on the cluster level with *P* < 0.05.

The contrast between complex OSV structure sentences and easy SOV structure sentences allowed identifying brain regions that are involved in the processing of syntactic complexity (see Fig. 4). There was increased activation for OSV sentences compared with SOV sentences in a left-lateralized network including the IFG pars opercularis (BA44; peak MNI coordinate xyz = −54, 11, 5; *P*_FWE_ < 0.001, *t* = 6.16, *k* = 360), preSMA (peak MNI coordinate xyz = −3, 8, 2; *P*_FWE_ < 0.001, *t* = 7.32, *k* = 260), premotor cortex (peak MNI coordinate xyz = −42, 2, 53; *P*_FWE_ = 0.002, *t* = 5.38, *k* = 200) and posterior middle temporal gyrus (peak MNI coordinate xyz = −54, −40, 2; *P*_FWE_ = 0.045, *t* = 4.38, *k* = 93). These regions, especially the IFG and the posterior temporal gyrus, have been previously reported for syntactic processing (Friederici et al. 2003b; Friederici 2011; Segaert et al. 2012). Thus, the reported brain areas are sensitive to the increased processing demands of the noncanonical OSV structure.

#### Interaction Effect: Syntax by Speaker

**Figure 5:**
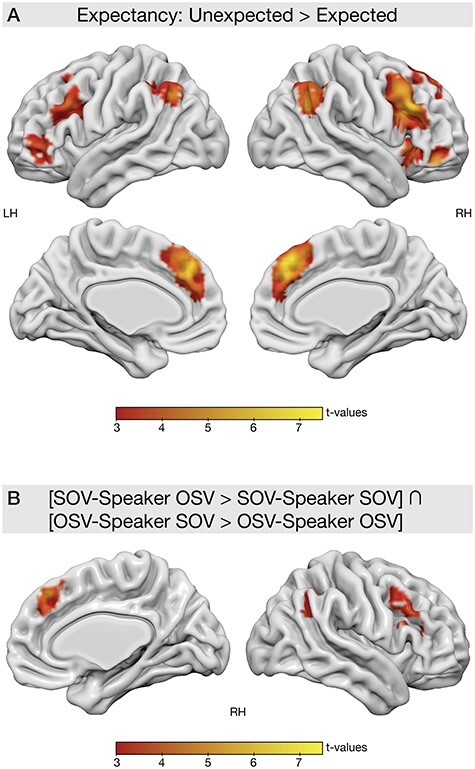
(*A*) Interaction effect of expectancy. The syntactic structure that was expected due to speaker-syntax coupling was contrasted to the unexpected syntactic structure for both speakers, respectively: [SOV-Speaker OSV + OSV-Speaker SOV] > [SOV-Speaker SOV + OSV-Speaker OSV]. Note that both conditions (i.e., expected and unexpected) contained the same amount of SOV and OSV structures, respectively. (*B*) Conjunction analysis of the contrasts [SOV-Speaker OSV > SOV-Speaker SOV] and [OSV-Speaker SOV > OSV-Speaker OSV]. Significant clusters are plotted on the brain surface. All results are FWE-corrected on the cluster level with *P* < 0.05.

The experimental paradigm allowed to investigate the effects of expectancy processing independently of syntactic complexity processing (Fig. 5). For that reason, the unexpected syntactic structure, regardless of speaker, was contrasted against the expected syntactic structure [(SOV-Speaker OSV + OSV-Speaker SOV) > (SOV-Speaker SOV + OSV-Speaker OSV)]. The results revealed activation in a bilateral fronto-parietal network with a dominance of the right hemisphere. Increased activation for unexpected compared with expected structures was found in bilateral preSMA (peak MNI coordinate xyz = 6, 35, 47; *P*_FWE_ < 0.001, *t* = 7.22, *k* = 1862) extending into the right MFG (peak MNI coordinate xyz = 42, 17, 44) and right IFG pars triangularis (peak MNI coordinate xyz = 45, 23, 29), bilateral angular gyrus (left peak MNI coordinate xyz = −48, −58, 41; *P*_FWE_ < 0.001, *t* = 5.39, *k* = 367; right peak MNI coordinate xyz = 51, −55, 32; *P*_FWE_ < 0.001, *t* = 6.27, *k* = 446) and bilateral IFG pars orbitalis (BA47; left peak MNI coordinate xyz = −33, 29, 17; *P*_FWE_ < 0.001, *t* = 4.80, *k* = 610; right peak MNI coordinate xyz = 45, 41, −16; *P*_FWE_ < 0.001, *t* = 6.06, *k* = 371). It has to be noted that due to the nature of the experimental design, trial numbers were not balanced across conditions in the analysis of the interaction (see Table 1). Therefore, the analysis was repeated using a set of matched trials for all conditions. Importantly, this analysis revealed similar activation clusters (see Supplementary Figs S1–S5).

In order to investigate whether the mismatch effect was driven by only one of the speakers (e.g., the unexpected OSV structure for the SOV-Speaker), a conjunction analysis was conducted using the 2 contrasts where the unexpected structure was contrasted against the expected structure for both speakers: SOV-Speaker OSV > SOV-Speaker SOV and OSV-Speaker SOV > OSV-Speaker OSV (see Fig. 4). The conjunction analysis revealed activation clusters only in the right hemisphere: the preSMA (peak MNI coordinate xyz = 42, 17, 44 *P*_FWE_ = 0.004, *t* = 4.28, *k* = 215), the MFG extending into the IFG pars triangularis (peak MNI coordinate xyz = 42, 17, 44; *P*_FWE_ = 0.004, *t* = 4.28, *k* = 215) and the angular gyrus (peak MNI coordinate xyz = 42, 17, 44; *P*_FWE_ = 0.004, *t* = 4.28, *k* = 215). This analysis highlights fronto-parietal structures especially in the right hemisphere as neural substrates for speaker-specific expectancy processing.

Taken together, the presented analyses provide information on the neural processes that take place when there is a mismatch between the actual syntactic structure of a sentence and the syntactic structure that is expected due to speaker identity, independent of syntactic complexity.

### Univariate Analysis: Probe Trials

**Figure 6:**
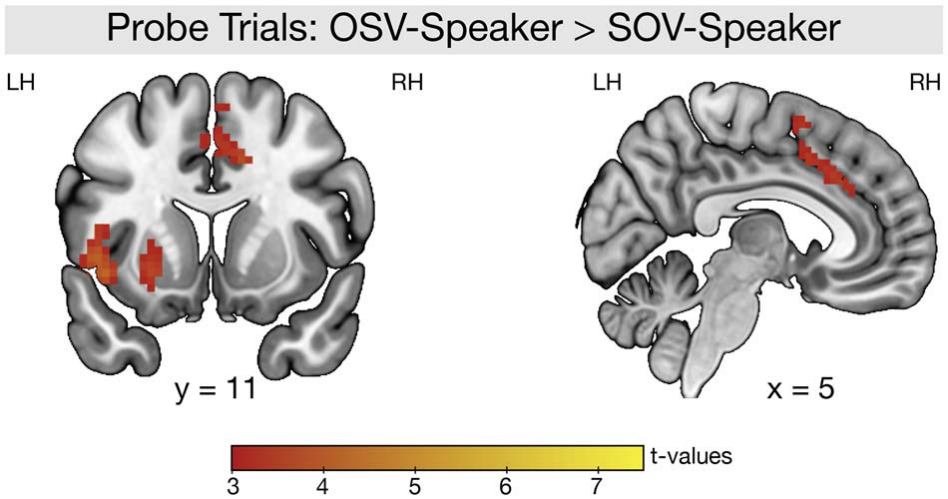
Speaker effect in the probe trials where the critical determiners were replaced by noise. The data show significant activation for the OSV-Speaker compared with the SOV-Speaker in the probe trials. In these trials, no actual syntactic structure information was provided in the sentences. All results are FWE-corrected on the cluster level with *P* < 0.05.

The ambiguous probe trials gave the unique opportunity to investigate top-down expectations about syntactic structures. Probe trials of the OSV-Speaker were contrasted against probe trials of the SOV-Speaker (Fig. 6). The contrast OSV-Speaker vs. SOV-Speaker revealed increased activation for the OSV-Speaker in a cluster involving the left Insula (peak MNI coordinate xyz = −42, 11, 7; *P*_FWE_ = 0.008, *t* = 4.59, *k* = 262) and the left Putamen (peak MNI coordinate xyz = −24, 20, −4) and a cluster involving the left anterior cingulate cortex (peak MNI coordinate xyz = −9, 17, 23; *P*_FWE_ = 0.004, *t* = 4.28, *k* = 215) and the right pre-SMA (peak MNI coordinate xyz = 12, 14, 38). There were no significant activation clusters for the reversed contrast (SOV-Speaker > OSV-Speaker). The analysis of the probe trials thus revealed an influence of cortical and subcortical areas in the processing of top-down speaker-specific syntactic expectations.

## Discussion

The present study was successful in inducing speaker-specific syntactic expectations, that is, listeners learned that one speaker preferred an easy sentence structure and the other a more complex sentence structure. In a next step, we tested how these expectations affect the neural response toward easy and complex structures. Importantly, we observed a differentiation between processing of general language priors related to the notion of syntactic complexity and individual speaker-specific expectation processing.

Syntactic complexity was observed in a left lateralized fronto-temporal brain network. These areas have previously been reported to relate to syntactic processing (Just et al. 1996; Bornkessel et al. 2005; Newman et al. 2010; Ben-Shachar et al. 2013; Meyer and Friederici 2015). As discussed earlier, most of these studies confounded syntactic complexity with their frequency of occurrence. The present results, however, demonstrate that our effect of syntactic complexity is not just a mere effect of a speaker’s frequency to use a particular structure (in a sense that OSV structures are less frequent) because the syntactic network was activated independent of speaker. It needs to be acknowledged, however, that all participants were native German speakers and therefore had a lifetime of exposure to these syntactic structures. The observed effects might therefore still reflect frequency differences, albeit on a population and not on a speaker level. This indicates that left fronto-temporal brain areas including the left IFG pars opercularis and the posterior temporal cortex probably underlie some very basic processing of syntactic structure, that is activated whenever syntactic structure needs to be reordered and hierarchical relations need to be processed (Bornkessel-Schlesewsky et al. 2012; Jeon 2014). These brain structures are therefore candidates to represent language priors related to syntactic structure that are formed by lifelong language use.

The actual speaker-specific processing of syntactic structure was observed in a different network, namely a right-hemispheric network of fronto-parietal regions. These regions showed increased activation when a speaker produced a sentence that was not expected based on the speaker’s language preferences, i.e., a mismatch. Therefore, this network might support a matching between speaker expectations and actual input. This is also supported by studies that show increased activation in right frontal areas when a sentence mismatches a discourse context (Kuperberg et al. 2000; Menenti et al. 2009), suggesting a comparison of contextual expectations and input. Furthermore, a mismatch between expectation and input might indicate that the internal model, which was used to generate the expectations, needs to be updated. A mismatch might also indicate high processing demands with respect to cognitive control. Such processing has been proposed by a model on top-down attention that reports fronto-parietal activation in response to perceptual deviant processing and increased cognitive control (Downar et al. 2000; Corbetta and Shulman 2002). It is therefore likely, that the observed processes are not exclusive to speaker-specific language expectations but might be domain general (Fedorenko et al. 2013).

Besides the dissociation between syntactic core processing and speaker-specific expectations observed in the present study, previous findings in the literature suggest a top-down influence of speaker information on language comprehension (Lattner and Friederici 2003; Van Berkum et al. 2008; Hanulíková et al. 2012). In the present study, this top-down influence was investigated using the ambiguous probe sentences. When comparing the complex speaker with the easy speaker, we observed activation in left fronto-striatal brain regions. This contrast demonstrates how top-down information of speaker-specific language preferences influence language processing that is based on more general language priors. In case of the OSV-speaker, the syntactic expectation that is generated due to speaker identity (i.e., the expectation for an OSV structure) is in contrast to the general language distribution of syntactic structures and thus might require additional control mechanisms. Striatal brain areas, namely the putamen, support this process, as they have been related to controlled syntactic processing in previous studies (Friederici and Kotz 2003; Friederici et al. 2003a; Stowe et al. 2004). Activation in these areas might relate to the inhibition of the generally preferred SOV structure in favor of the alternative OSV structure that is only expected due to speaker identity (Mestres-Missé et al. 2012, 2014). Please note, that the experimental task in the probe trials was explicit, as it required participants to directly indicate their interpretation of the sentence. It is therefore possible that participants engaged in an explicit processing of speaker-specific expectations. While we cannot differentiate between explicit and implicit learning in our present experiment, it should be acknowledged that previous studies have observed effects of language exposure also with more implicit measures (Kamide 2012; Fine et al. 2013).

Furthermore, an additional cross-classification analysis was conducted in order to test whether brain regions represent syntactic structure information on the basis of speaker identity in the probe trials. There was higher-than-chance decoding accuracy in the frontal pole as well as medial frontal and parietal lobe (see Supplementary Figs S1–S5). These areas have been related to social cognition (Saxe and Kanwisher 2003; Van Overwalle 2009; Frith and Frith 2012; Bludau et al. 2014). This finding suggests that participants held representations of speaker-identity that also encoded information on preferential syntactic language use. In line with the univariate results, there was no differentiation in brain regions related to syntactic complexity processing, suggesting that speaker-specific expectations do not directly involve core syntactic processing.

Our findings demonstrate an important neural mechanism that allows a listener to represent general language principles while retaining the ability to adapt to speaker-specific demands. Speaker-specific information thus involves top-down processing to meet the demands of a (complex) expected syntactic structure. Furthermore, syntactic information became reflected in brain areas related to social cognition, possibly reflecting “enriched “representation of speaker-identity.

The present results show that listening to a single speaker, whose language use contrasts a population-wide language distribution, does not change the way in which the brain processes language in general. Yet, a couple of these sentences are enough to form a speaker-specific representation that is highly sensitive to deviants (and is present even after 9 months as behavioral data suggest, Kroczek and Gunter 2017). Furthermore, this newly acquired knowledge directly affects the language comprehension system via controlled processing. As this process takes place within 2 days with only a few minutes of exposure to the speakers, one could speculate that a lifelong exposure to different speakers with different patterns of language use might help to generate population-wide representations of language use (cf. MacDonald 2013; Kuperberg and Jaeger 2015). Previous studies have demonstrated adaptation of expectations after exposure to a particular language input (Fine et al. 2013; Fraundorf and Jaeger 2016; Ryskin et al. 2017), and it has been demonstrated that speakers start to use previously unknown language variations after exposure (Fanselow et al. 2005). These representations might ultimately form basic language principles and could be used to generalize to new speakers, when no information about language use is available (Kleinschmidt and Jaeger 2015).

In sum, we show that the human brain immediately adapts to speaker-specific language use and implements this top-down information in language processing, while general language principles remain represented in the language circuit. This might constitute a neural mechanism for dealing with both interindividual differences and general, population-wide language priors at the same time.

## Notes

We are grateful to Angela D. Friederici for her kind support of the research described here and her helpful discussions during the preparation of this paper. We thank Nicole Pampus for data acquisition and Kerstin Flake and Andrea Gast-Sandmann for their help in assembling the figures. *Conflict of Interest*: None declared.

## Supplementary Material

SupplementaryMaterial_tgaa021
