# Supplementary material for: Distinct Neural Networks Relate to Common and Speaker-Specific Language Priors
Source: Cereb Cortex Commun. 2020 May 29;1(1):tgaa021. doi: 10.1093/texcom/tgaa021 (PMC8153046; doi:10.1093/texcom/tgaa021)
Supplement: SupplementaryMaterial_tgaa021 [file supplementarymaterial_tgaa021.docx]

**Supplementary material**

***Incremental build-up of syntactic expectations in the exposure phase***

In order to investigate how expectations were adapted over the course of the sessions, a growth curve analysis was conducted on participants’ responses in the probe trials that were interleaved in the exposure phase (Mirman, 2014). The empirical logit of SOV responses in non-overlapping windows of four trials was taken as the dependent variable in a linear mixed-effects model. The model included fixed effects for the factors *Speaker* (sum coded: SOV-Speaker = 1, OSV-Speaker = -1), *Session* (sum coded: Session 1 = 1, Session 2 = -1) and *Time* (continuous regressor). The random effects were defined on the subject level and on the lowest level of nesting in the data (both allowing for random slopes for Time).

The growth curve analysis revealed a main effect of *Speaker* [χ^2^ (1) = 109.292, p < .001], an interaction of *Speaker x Time* [χ^2^ (1) = 9.385, p = .002], an interaction of *Speaker x Session* [χ^2^ (1) = 12.758, p < .001] and an interaction of *Speaker x Time x Session* [χ^2^ (1) = 5.638, p = .018]. The parameter of the *Speaker x Time* interaction revealed a linearly increasing difference in syntactic choices due to speaker over the course of the sessions [$\hat{\beta}$ = 0.034, t(112) = 3.064, p = .002]. The *Speaker x Session* interaction demonstrated an increased speaker effect when comparing the fMRI Session to the training Session [$\hat{\beta}$ = -0.371, t(112) = -4.220, p < .001]. Furthermore, the three-way interaction of *Speaker x Time x Session* indicated a stronger linear increase of the speaker effect in the training session compared to the fMRI session [$\hat{\beta}$ = 0.026, t(112) = 2.375, p = .019].

In summary, these results demonstrate a dynamic adaptation of expectations to the two speakers over the course of the experiment. This adaptation was predominant in the training session, while the effects were maintained in the test session (Figure S1). An unsmoothed picture of the data is presented in Figure S2, which shows the percentage of participants, who expected a SOV structure at single probe trials for both speakers and sessions.


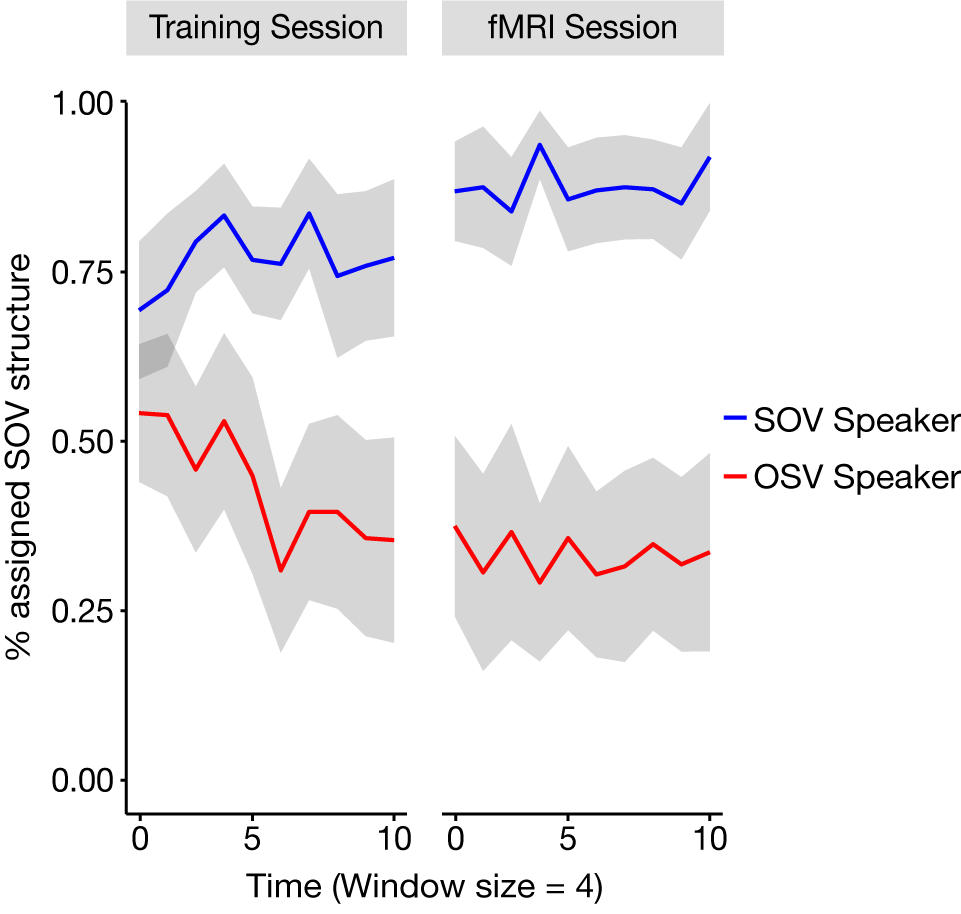


*Figure S1: Percentage of assigned SOV structures in the exposure phase. The figure shows the percentage of trials for which the SOV structure had been assigned to a probe sentence during the exposure phase in both sessions. The data were subdivided into non-overlapping windows containing 4 trials per speaker (leading to 10 time points per session). For every window the proportion of ’SOV’ responses was calculated. The shaded areas depict 95% confidence intervals over the means of participants.*

*
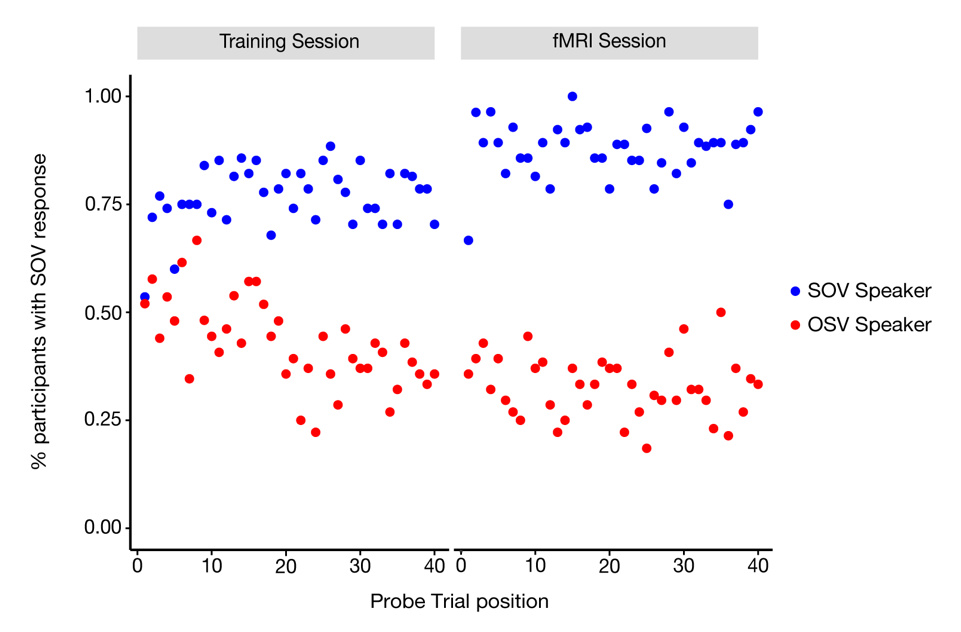
*

*Figure S2: Percentage of participants who chose a ‘SOV’ response in the probe trials within and across the training session and the fMRI session for both speakers. Probe trial position on the x-axis is ordered with respect to the position in the session, i.e. position 1 refers to the first probe trial in a particular Speaker condition and so forth.*

***Speed-accuracy trade-off***

In order to investigate whether the differences in error rates between sessions and between syntactic structures can be explained by reaction times, we analyzed participants’ response (correct vs. incorrect) in a logit mixed-effect model that included reaction times. This model included fixed effects fixed effects for the factors *Structure* (sum coded: SOV = 1, OSV = -1) and *Session* (sum coded: Training session = 1, fMRI session = -1) as well as log-transformed *Reaction Times* and interactions of all three predictors. The model also included random intercepts for subjects and items as well as random slopes by subject for Structure, Session, and for the interaction of both.

The model of participants' responses revealed a main effect of *Structure,* χ^2^ = 64.874, p < .001, with increased error rates for the OSV sentences compared to SOV sentences ( = -2.474, SE = 1.444), a main effect of *Session,* χ^2^= 40.77, p < .001, with decreased error rates in the fMRI session compared to the training session ( = 3.096, SE = 1.486), and a main effect of *Reaction Times,* χ^2^= 236.575, p < .001, with higher reaction times being related to increased error rates ( = 2.272, SE = 0.202). There were no significant interaction effects. These data suggests a general influence of reaction times on task accuracy. Participants answered less accurate when they took more time to answer. Importantly, there were also additional effects of syntactic structure and session, demonstrating that OSV structures were more difficult than SOV structure. Furthermore, there was improvement in task performance from the training Session to the fMRI session. These data suggest a real performance improvement, rather than a speed-accuracy trade-off.


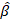

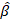

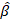

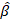

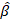

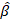


***Main effect of speaker identity in regular trials***

The main effect of speaker identity was analyzed by contrasting sentences that had been produced by the OSV-Speaker to sentences that had been produced by the SOV-Speaker (Figure S3, there was no significant activation for the reversed contrast). Significant clusters were elicited in the bilateral right superior temporal gyrus (left peak MNI coordinate xyz = -54, -34, 8; P_FWE_=.024, t = 4.47, k = 112; right peak MNI coordinate xyz = 60, -10, -1; P_FWE_<.001, t = 5.36, k = 503), the left putamen (peak MNI coordinate xyz = -18, 5, 5; P_FWE_<.001, t = 6.29, k = 4612) extending into the left MFG (peak MNI coordinate xyz = -39, 32, 29) and left anterior Insula (peak MNI coordinate xyz = -24, 20, 8), the right cerebellum (peak MNI coordinate xyz = 27, -67, -28; P_FWE_<.001, t = 5.05, k = 1198) extending into the fusiform gyrus (peak MNI coordinate xyz = 36, .55, 31), bilateral precuneus (left peak MNI coordinate xyz = -12, -55, 50; right peak MNI coordinate xyz = 6, -64, 53; P_FWE_=.002, t = 4.59, k = 197) and bilateral IPL (left peak MNI coordinate xyz = -36, -46, 41; P_FWE_<.001, t = 4.93, k = 560; right peak MNI coordinate xyz = 36, -46, 50; P_FWE_<.001, t = 4.56, k = 295). This contrast shows increased neural activation for the speaker that was associated the complex syntactic structure in comparison to the speaker that was associated with the easy syntactic structure.

*
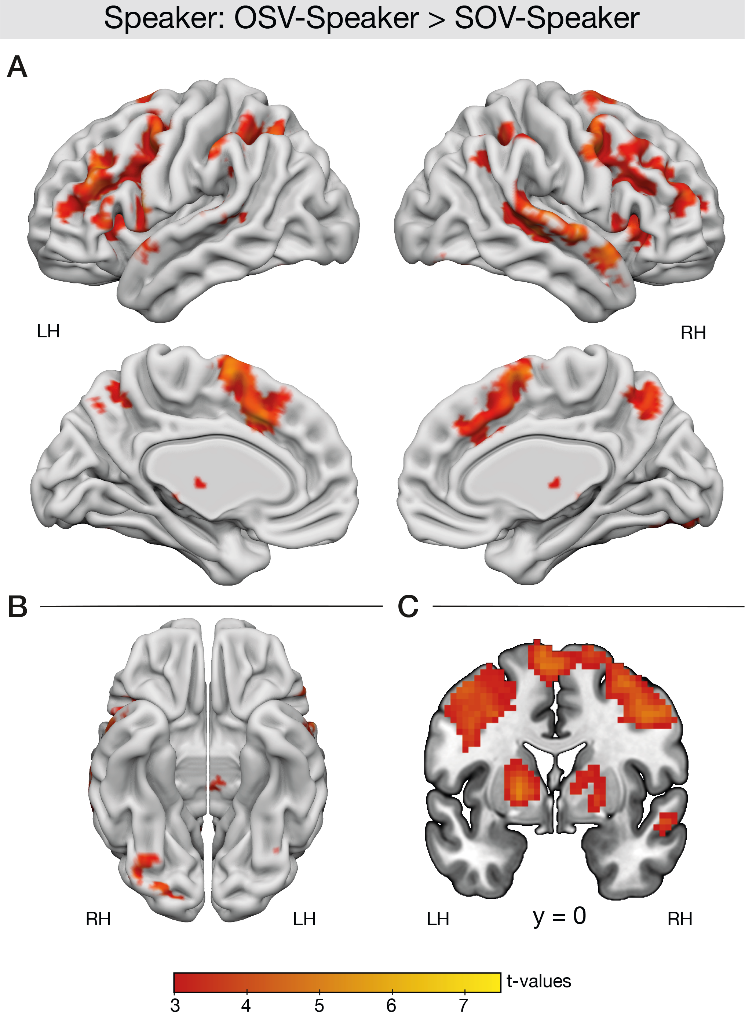
*

*Figure S3: Main effect of speaker identity in the regular sentence trials. Neural activation was increased for OSV-Speaker trials compared to SOV-Speaker trials. Figure A shows significant clusters on the brain surface. Figure B shows the brain surface with an inferior view. Figure C shows subcortical activation on a coronal slice of the brain. All results are FWE-corrected on the cluster level with p < .05.*

***Analysis of the interaction effect with matched trial numbers***

The present paradigm involved different trial number in different conditions in order to establish expected and unexpected syntactic structures for a particular speaker (overall trial numbers of syntactic structures and speakers were balanced). In order to test whether different trial number might have an effect on the interaction effect when contrasting unexpected vs. expected syntactic structures for the particular speakers, we performed the same analysis while only including a random and reduced set of trials in the expected conditions (i.e. SOV sentences for the SOV-speaker and OSV sentences for the OSV-speaker). Thereby all conditions were entered with 40 trials in the analysis. Importantly, similar results were obtained as in the initial analysis (see Figure S4). There was increased activation in the bilateral preSMA (left peak MNI coordinate xyz = -24, 23, 53; right peak MNI coordinate xyz = 6, 35, 47; P_FWE_<.001, t = 6.19, k = 561), the bilateral MFG (left peak MNI coordinate xyz = 36, 29, 20; P_FWE_=.001, t = 4.40, k = 191; right peak MNI coordinate xyz = 42, 23, 32; P_FWE_=.001, t = 5.16, k = 603), the right anterior Insula (peak MNI coordinate xyz = 36, 23, -4; P_FWE_=.039, t = 4.30, k = 90), bilateral angular gyrus (left peak MNI coordinate xyz = -48, -58, 41; P_FWE_<.001, t = 5.07, k = 251; right peak MNI coordinate xyz = 51, -55, 29; P_FWE_<.001, t = 5.21, k = 276) as well as a trend towards a significant cluster in the left IFG pars orbitalis (peak MNI coordinate xyz = -36, 47, -13; P_FWE_=.085, t = 4.30, k = 69). There were also some differences in comparison to the full analysis. For instance, activation did not extend in the right IFG pars triangularis and IFG pars orbitalis. Instead we observed activation in the right anterior Insula. We believe that these differences are related to different trial numbers and possibly reflect inconsistent effects. Importantly, activation in the bilateral MFG, preSMA and angular gyrus was present in both analysis. These regions were additionally highlighted in the conjunction analysis (see Figure 5). These results suggest that the observed interaction effect between syntactic structure and speaker identity does not result from different trial numbers in the main analysis.

*
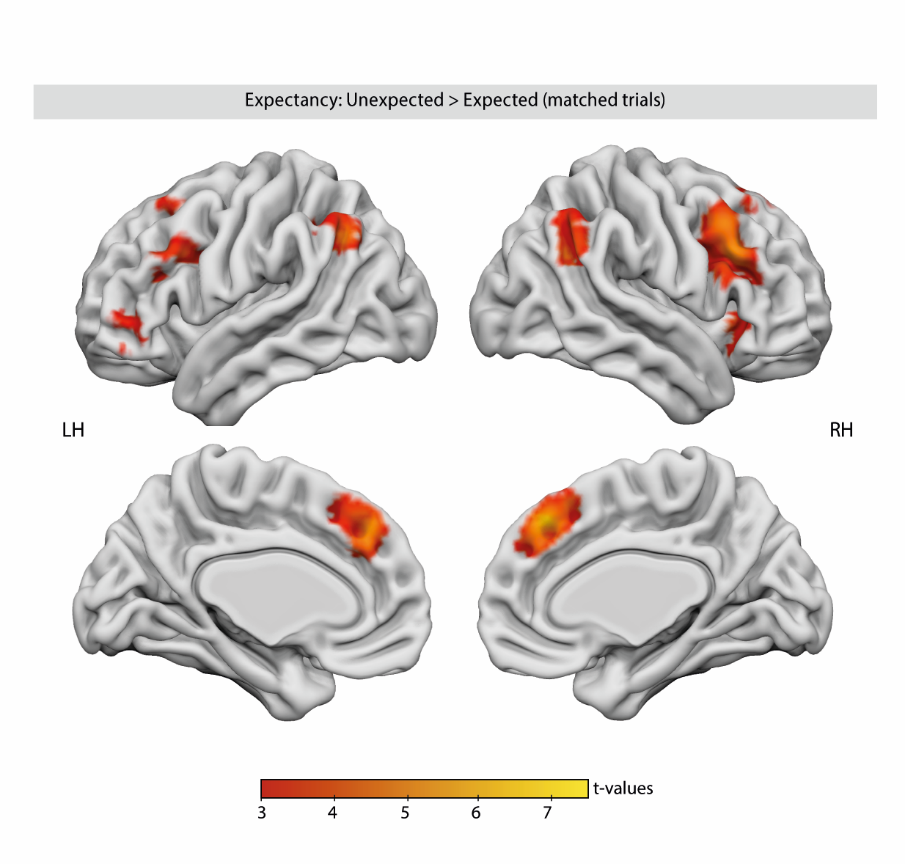
*

*Figure S4: Interaction effect of expectancy with matched trial numbers between conditions. The syntactic structure that was expected due to speaker-syntax coupling was contrasted to the unexpected syntactic structure for both speakers respectively: [SOV-Speaker OSV + OSV-Speaker SOV] > [SOV-Speaker SOV + OSV-Speaker OSV]. Note, that both conditions (i.e. expected and unexpected) contained the same amount of SOV and OSV structures respectively. Significant clusters are plotted on the brain surface. All results are FWE-corrected on the cluster level with p < .05.*

***Multivariate analysis: Cross-classification***

A central goal of the present experiment was to investigate whether listeners would represent syntactic information about sentence structure on the basis of speaker identity. For that reason, we implemented the probe trials where SOV/OSV structure information was not presented in the sentence stimulus due to a white noise manipulation carried out on the articles but speaker information was present. Importantly, univariate and multivariate methods may reveal different aspects of the data: In our cases, univariate analysis methods are useful to reveal differences in processing related to speaker identity. Crucially, these differences can arise not only from different information but also due to different task demands. Multivariate methods, however, can reveal similarities in information content across different conditions (Kaplan, Man, & Greening, 2015). Therefore, we conducted a cross-classification analysis using the Decoding Toolbox (Hebart, Görgen, & Haynes, 2015) where we trained a classifier to differentiate between syntactic structure in the regular trials using a support vector machine. Importantly, SOV and OSV trials consisted equally of SOV-Speaker and OSV-Speaker trials, this means that in case of the [SOV-Speaker SOV] and [OSV-Speaker-OSV] conditions, we randomly selected trials in order to match the numbers in the [SOV-Speaker OSV] and [OSV-Speaker SOV] conditions respectively. Then, we tested this classifier on the probe trials in order to investigate which brain regions would correctly differentiate between SOV-Speaker and OSV-Speaker. This analysis allowed to test whether syntax information is represented solely on the basis of speaker identity. A searchlight approach using a sphere of 12 mm on the whole brain was used for every participant. The resulting brain images showed classifier accuracy minus chance for every voxel. These data were entered in second-level analysis in SPM using a one-sample t-test. Results were FWE corrected for multiple comparisons on a cluster-level using a cluster-forming threshold of p <.001 and a FWE threshold of p < .05.

The results revealed better-than-chance decoding accuracy in clusters in frontal, temporal parietal brain regions, namely the right frontal pole (peak MNI coordinate xyz = 21, 62, 5; P_FWE_ < .001, t = 6.05, k = 61), the right superior frontal gyrus (SFG) (extending into the MFG) peak MNI coordinate xyz = 21, 35, 41; P_FWE_ < .001, t = 5.68, k = 134), left medial anterior temporal lobe (peak MNI coordinate xyz = -24, 5, -22; P_FWE_ < .001, t = 5.24, k = 60), left superior medial gyrus (peak MNI coordinate xyz = -21, 26, 32; P_FWE_ < .001, t = 5.24, k = 84), right precuneus (peak MNI coordinate xyz = 12, -55, 62; P_FWE_ = .002, t = 4.65, k = 40) , right angular gyrus (peak MNI coordinate xyz = 30, -49, 38; P_FWE_ = .024, t = 4.25, k = 25) as well as left Cerebellum (peak MNI coordinate xyz = -27, -52, -34; P_FWE_ = .024, t = 4.12, k = 25). Brain activation in these areas allowed to differentiate between speakers on a better-than-chance level using a classifier trained on syntactic information (Figure S5). Therefore, the results show neural substrates which represent syntactic information on the basis of speaker identity.

***
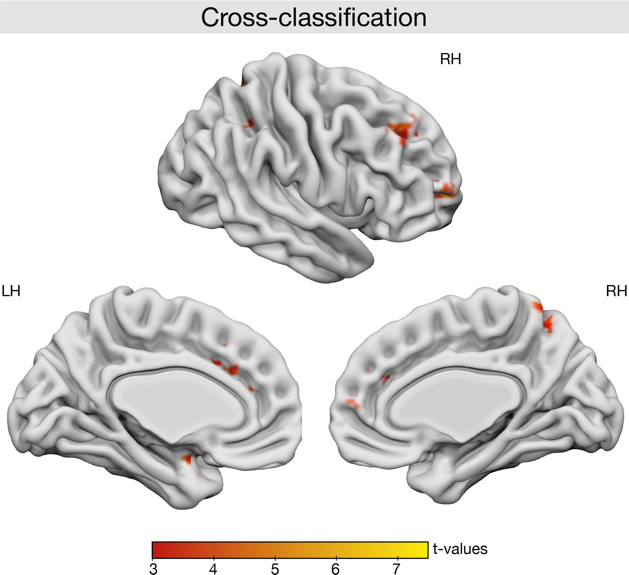
***

*Figure S5: Results of the cross-classification analysis where a classifier, which was trained to differentiate between SOV and OSV sentences in the regular trials, was tested to differentiate between SOV-Speaker and OSV-Speaker in the probe trials. Results depict better-chance-accuracy on the group level. All results are FWE-corrected on the cluster level with p < .05.*

***References***

Hebart, M. N., Görgen, K., & Haynes, J. D. (2015). The decoding toolbox (TDT): A versatile software package for multivariate analyses of functional imaging data. *Frontiers in Neuroinformatics*, *8*(JAN), 1–18. https://doi.org/10.3389/fninf.2014.00088

Kaplan, J. T., Man, K., & Greening, S. G. (2015). Multivariate cross-classification: Applying machine learning techniques to characterize abstraction in neural representations. *Frontiers in Human Neuroscience*, *9*(MAR), 1–12. https://doi.org/10.3389/fnhum.2015.00151

Mirman, D. (2014). *Growth Curve Analysis and Visualization Using R*. Boca Raton, FL: CRC Press.
